# Using survival prediction techniques to learn consumer-specific reservation price distributions

**DOI:** 10.1371/journal.pone.0249182

**Published:** 2021-04-29

**Authors:** Ping Jin, Humza Haider, Russell Greiner, Sarah Wei, Gerald Häubl

**Affiliations:** 1 Department of Computing Science, University of Alberta, Edmonton, Canada; 2 Alberta Machine Intelligence Institute, Edmonton, Alberta, Canada; 3 Warwick Business School, the University of Warwick, Coventry, United Kingdom; 4 Alberta School of Business, University of Alberta, Edmonton, Canada; Roswell Park Cancer Institute, UNITED STATES

## Abstract

A consumer’s “reservation price” (RP) is the highest price that s/he is willing to pay for one unit of a specified product or service. It is an essential concept in many applications, including personalized pricing, auction and negotiation. While consumers will not volunteer their RPs, we may be able to predict these values, based on each consumer’s specific information, using a model learned from earlier consumer transactions. Here, we view each such (non)transaction as a *censored observation*, which motivates us to use techniques from survival analysis/prediction, to produce models that can generate a consumer-specific RP distribution, based on features of each new consumer. To validate this framework of RP, we run experiments on realistic data, with four survival prediction methods. These models performed very well (under three different criteria) on the task of estimating consumer-specific RP distributions, which shows that our RP framework can be effective.

## 1 Introduction

### 1.1 Motivation

Reservation price (RP) is the highest price a consumer is willing to pay for one unit of a certain product or service [1], which is an important and widely used concept in both the economics and marketing literature. It is critical for designing various pricing strategies, such as personalized pricing [2, 3], one-to-one promotion [4], and optimal pricing [5]. Many other fields, including auction [6, 7], ad exchange [8], negotiation, and the design and pricing of bundles [9, 10], also heavily rely on accurate estimations of consumers’ RPs.

**Fig 1:**
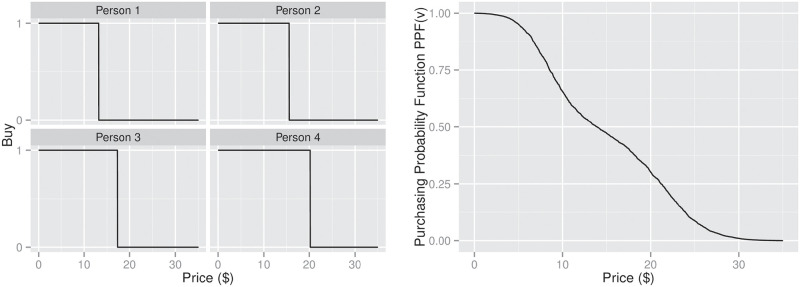
The overall purchasing probability function–(left) for 4 individuals; (right) over a population.

For example, suppose that we are interested in setting the price of a certain product *ω* to achieve maximum profit when selling it to a certain population. If we know the reservation price *r*_*i*_ of each subject *i*, then we can easily compute the overall purchasing probability function *PPF*(*v*) over price *v* for this specific population as

$$\begin{array}{c} PPF\left(v\right)\;=\;\frac{1}{n}\sum_{i}^{n}I\left\{v\;\leq \;r_{i}\right\}\;, \end{array}$$ (1)

where *n* is the total number of consumers and **I**{*ϕ*} = 1 if the proposition *ϕ* is true, and 0 otherwise; see Fig 1.

If we knew *PPF*(*v*), we could achieve the maximum expected profit by setting the price of *ω* to be

$$\begin{array}{c} v^{*}\;=\;\underset{v}{arg max}\left\{\left(v-c\right)\cdot PPF\left(v\right)\right\}, \end{array}$$ (2)

where *c* is the production cost of *ω*. (Throughout, we use the term “production cost” to mean the incremental cost of producing a single unit).

Moreover, if we are allowed to sell *ω* at different prices to different subjects–*i.e.*, first degree price discrimination [11]–then the seller’s best strategy for maximum profit is to sell *ω* to the subjects at their individual reservation prices–*i.e.*, using Fig 1, sell to Person 1 at $13.20, sell to Person 2 at $15.60, etc. (here, we assume the production cost is under $10).

In the scenario of e-commerce, which has enjoyed booming development recently, the online retailers also have great interest in designing pricing strategies, understanding consumers’ purchasing decisions, doing one-to-one promotion and so on, which rely on accurate estimation/elicitation of consumers’ RPs. Additionally, online retailers usually have more information available than the traditional offline ones about their consumers, such as consumer-specific information (demographics, this consumer’s historical transactions and so on) and historical transactions of other consumers, which may be related to consumers’ RPs. This motivates us to find ways to better estimate consumers’ RPs with this available information.

### 1.2 Contributions

In this paper, we propose a novel framework of formulating the RP estimation problem, which involves explicitly defining a consumer’s RP as a random variable conditional on the consumer’s features [12]. This probabilistic framework not only captures the inherent uncertainty of RP, but also allows us to use stochastic models to express the relationship between RP and consumer-specific features.

We take a machine learning approach to this “RP estimation” task: inferring a probabilistic RP model from information about previous consumers—notably their transaction data (the observation that a consumer decided to “buy” a product at some specified price) and non-transaction data (the observation that a consumer intentionally decided to “not buy” a product at some specified price). Here, each non-transaction instance refers to an *explicit* decision to not purchase a product at the offered price–*e.g.*, when a consumer puts an item in the shopping cart or the wish list, but does not buy it.

We can then apply this learned model to a specific consumer, to produce that consumer’s specific posterior distribution over RP, and use it to predict whether a consumer is likely to purchase the product. Note this approach does not require individual consumers to directly report their RPs, and so avoids some of the problems associated with that alternative approach; these relate to the first two drawbacks described in Subsection 3.1.

Beyond these foundations, implementing this required three novel contributions: First, we note that *the purchasing (resp., non-purchasing) observations correspond to right censored (resp., left censored) observations in the survival analysis setting*, which motivates us to *utilize various survival techniques to learn a model* that maps the features of a consumer to his/her RP, from historical (non-)transaction data—note the term “(non-)transaction data” refers to both transaction data and non-transaction data. (We discuss below how this relates to other marketing/auction results that view earlier data as being censored). Second, we provide *empirical evidence* (using several appropriate datasets) that this framework is effective in producing consumer-specific pricing, which can lead to greater profit than fixed pricing. Third, we introduce a fairly new learning tool for survival analysis, multi-task logistic regression (MTLR), to the marketing community, and demonstrate that this MTLR system is competitive–either outperforming or matching many standard tools, across three different measures.

This work is relevant to the marketing community as it means that a seller can first learn an RP-model for a specific product, based only on data that is often readily available–the (non-)transaction logs, along with some consumer description data–then apply the resulting model to accurately estimate the individual RP distribution of that product for a novel consumer, even if that consumer has not bought the product of interest before, or even is completely new.

Note this paper focuses on the task of estimating a consumer’s reservation price, but not about how a seller would use that information. We briefly touch on this topic in Section 6.4.

### 1.3 Other marketing applications of survival analysis ideas

As a final preliminary comment, note that prior work has explored ways to use ideas from survival analysis to tackle a variety of other marketing phenomena. For background, Hosmer et al. [13] provides a nice general introduction to survival analysis in general, and Wang et al. [14] summarizes many machine learning techniques and evaluation metrics for survival analysis. One obvious example is predicting when a customer will cease his/her relationship with a company–this is called “customer attrition” or “customer churn.” This corresponds exactly to survival analysis, as it is predicting the time to an event, where (right) censoring means a consumer is still with the company [12, 15]. The present work relates in terms of censoring of items, but differs as it deals with price, rather than time; it also considers both left- and right-censoring, etc.; Table 2 shows the connections. We note that some project, including [16, 17], connect this time to cost: Given that the treatment cost for a patient accumulates over time, if the study ends before the treatment is complete for a patient (or when that patient is lost to follow-up), we will not know his/her total cost–*i.e.*, that person’s lifetime-medical-cost is left-censored. The present work differs as (1) our reasons for under-bounding the cost is not due to temporal truncation, but rather a model learned from earlier observations about (non)transactions of other consumers, being applied to a current consumer, which is (2) both left- and right-censored, and is (3) *personalized*, based on consumer features.

Ganchev *et al.* [18] also uses survival analysis techniques (here the Kaplan-Meier estimator) as a way to deal with the problem of order dispersion in “dark pools,” a relatively new kind of equities exchange in which traders seek to “invisibly” trade large volumes at market prices. Note that these applications are different from our goal, of predicting a consumer’s individual reservation price.

The present research is also similar to prior work on auctions: Blum *et al.* [19] observed that a bidder, at a sealed-bid auction, can use the auction outcomes to provide censored information about the other participants, which can be used to approximate their underlying bid distribution. Cesa-Bianchi *et al.* [20] and Amin *et al.* [21] use a similar observation in their analyses of second-price auctions–here, “only if we win the click do we observe the actual competing price; otherwise, we only know our bid was too low.” Our results differ by (1) considering individual consumer purchases, rather than winning multi-consumer auctions (and hence reservation *price*, rather than reservation *bid*), and (2) producing a model that involves learned non-linear combinations of consumer features (and so can estimate RPs of completely new consumers).

### 1.4 Outline

Section 3 describes our framework of RP estimation. Section 3.1 first summarizes the related literature, to place our work. Subsection 3.2 then introduces the formal definition of RP, and Subsection 3.3, the decision model that formulates the relationship between consumers’ purchasing decisions and their RPs. Subsection 3.4 illustrates our way to collect (non-)transaction data, which can be used to learn the RP distributions.

Section 4 first describes the relationship between the RP estimation problem and survival analysis problem. Then Subsection 4.2 introduces four survival models that can be used to estimate RPs: *viz.*, Kaplan-Meier Estimator, Cox proportional hazard model, accelerated failure time model, and the MTLR model.

Section 5 describes how we collected the needed data and some basic information about the four datasets. We also discuss several potential problems of data quality and ways to address them. (We used a survey to collect the relevant information; its questions appear in Appendix in S1 File).

Section 6 presents empirical results of using various survival models to estimate a consumer’s RP, under three different evaluation criteria: the mean absolute error of the RP predictions, the classification accuracy of predicting specific purchases, and estimating the profit obtained with a simple pricing strategy. All results are based on ten-times repeated ten-fold cross validation. The strong performance of these models in estimating consumer-specific RPs supports the effectiveness of our novel framework. This section also provides the features found to be most relevant to the prediction. Finally, Section 7 discusses three potential directions for future work, and Section 8 summarizes our contributions.

## 2 Methods

This study used the data about reservation price that we collected using a survey on Amazon Mechanical Turk. This study received written approval from the Research Ethics Office at the University of Alberta (Number: Pro00048923_REN1).

## 3 Framework of reservation price estimation

The common understanding of RP–*i.e.*, the highest price a consumer is willing to pay for a certain unit of product or service–indicates that consumers’ purchasing decisions on a certain product are closely related to their RPs of the product. Some methods (*e.g.*, BDM; see below) require actual purchasing to obtain accurate estimates of RPs. However, we may be able to avoid directly asking consumers to report their RPs, by instead inferring their RPs from their purchasing decisions (and the decisions of other consumers); this information is much easier to collect in practice. Therefore, in this section, we propose a consumer decision model that formulates the way consumers reach purchasing decisions and how it is related to their RPs. This decision model and a corresponding way of collecting data make up our framework of RP estimation. Within this framework, we can design new methods or utilize existing methods to learn the RP distributions from the observations of consumers’ purchasing decisions, *i.e.*, (non-)transaction data.

### 3.1 Previous analyses of reservation prices

**Fig 2:**
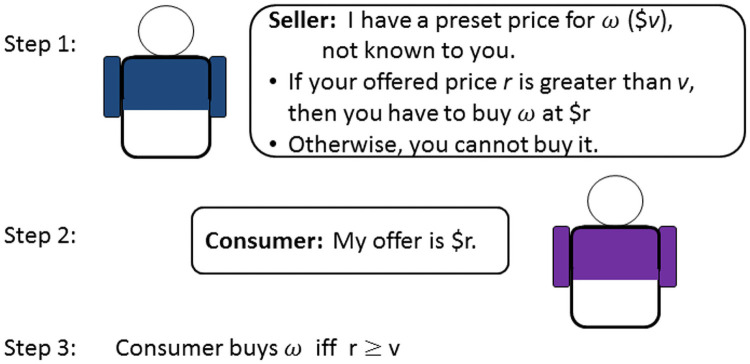
The Becker-DeGroot-Marschak method.

As revealing the true RPs will put consumers at a disadvantage in making deals with sellers, they would not voluntarily reveal this information. This had led to a huge amount of research efforts in designing incentive compatible methods for eliciting fixed-point RPs [6, 22–25]. In general, methods like the Becker-DeGroot-Marschak (BDM) method strive to make the consumers realize that revealing the true RP is their best strategy (see Fig 2), which is the key to accurate elicitation of consumers’ RPs.

However, it would be unrealistic to assume that a consumer’s RP for a product always stays the same. Wang *et al.* [25] also note that there is even uncertainty within an individual’s RP, due to the consumer’s uncertainty about his/her own preference [26] and the product performance [27].

**Fig 3:**
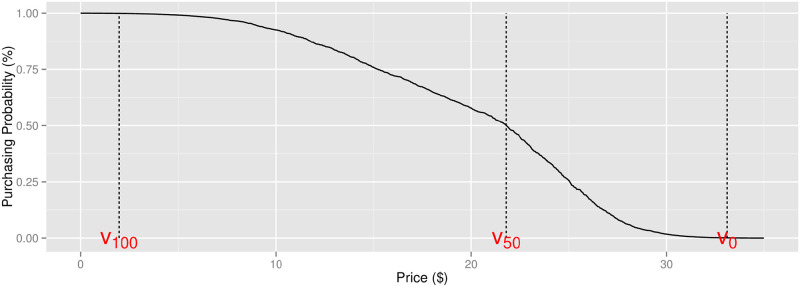
Three interpretations of reservation price: Floor RP, indifferent RP, and ceiling RP.

Therefore, several different interpretations of the RPs have been proposed [28–30], which are associated with different probabilities of purchasing (see Fig 3):

1. **Floor RP**: the maximum price at or below which the consumer will buy with 100% probability [28].
2. **Indifferent RP**: the price at which a consumer is indifferent between the money and the product—*i.e.*, s/he will buy it with 50% probability [29].
3. **Ceiling RP**: the minimum price at or above which the consumer will never buy it—*i.e.*, s/he has 0% probability of buying [30].

Furthermore, ICERANGE [25] embraced the inherent uncertainty of RP, by viewing a consumer’s RP as a price range instead of a single price point, which here means simultaneously eliciting several price points associated with different purchasing probabilities.

However, none of these methods deal with the challenge raised in the e-commerce scenario, *i.e.*, how to utilize the extra information in e-commerce to help the task of inferring consumers’ RPs. What is worse, they also suffer from several drawbacks, which make them ineffective in the e-commerce scenario:

1. Consumers have little patience and no motivation to participate in the elicitation activity.
2. It is hard to validate if consumers realize that their best strategy is to tell their true RPs, which may lead to inaccurate elicitation of RPs.
3. These methods have no capability of generalize beyond individual-level RP predictions–*i.e.*, each new consumer must go through the whole elicitation procedure to estimate his/her RP for the product of interest.

Therefore, we need a model that can overcome these drawbacks and can effectively utilize the new information available in e-commerce setting to help the task of estimating consumers’ RPs.

### 3.2 Stochastic setting of reservation price

These arguments motivate us to use a probabilistic interpretation of RPs [12]. Here, we let ${\vec{X}}_{}$ denote the random vector representing the features of consumers and ${\vec{x}}_{}$ denote a certain vector of feature values, corresponding to a single consumer. We also formally define two crucial random variables:

**Definition 1 (Consumer-specific reservation price)** *For a certain product ω*, *the consumer-specific RP* $R_{\omega }|{\vec{x}}_{}\;\in {ℜ}^{\geq 0}$ *is a random variable conditioned on the features of the consumer* ${\vec{X}}_{}={\vec{x}}_{}$.

**Definition 2 (Consumer-specific purchasing decision)** *If a product ω is offered at price v*, *the consumer-specific purchasing decision* $A_{\omega ,v}|{\vec{x}}_{}\in \left\{1,\;0\right\}$ *is a binary random variable, conditioned on the features of the consumer* ${\vec{X}}_{}={\vec{x}}_{}$. *(Note that* $A_{\omega ,v_{1}}|{\vec{x}}_{}$ *and* $A_{\omega ,v_{2}}|{\vec{x}}_{}$ *are two different random variables, for v*_1_ ≠ *v*_2_. *Also, by convention, we will identify the value 1 with “buy” and 0 with “not_buy”*).

### 3.3 Consumer decision model

In this section, we propose a decision-making model that describes how the consumer’s purchasing decision $A_{\omega ,v}|{\vec{x}}_{}$ is related to the RP $R_{\omega }|{\vec{x}}_{}$. When a consumer with features ${\vec{X}}_{}={\vec{x}}_{}$ is faced with a given offer–*i.e.*, a specific product *ω* is being offered at price *v*–s/he reaches her/his purchasing decision $a∼A_{\omega ,v}|{\vec{x}}_{}$ in a two-step procedure

**Step 1. Draw an “instant RP”**: an instant RP *r* is drawn from the distribution of $R_{\omega }|{\vec{x}}_{}$.
**Step 2. Make a decision**:
  $$\begin{array}{c} \text{Consumer}\;\text{will}\;\text{buy}\;\omega \;\text{for}\;\text{price}\;v\;\text{iff}\;v\leq r, \end{array}$$
  *i.e.*,
  $$\begin{array}{c} a=I\{v\leq r\}. \end{array}$$

**Fig 4:**
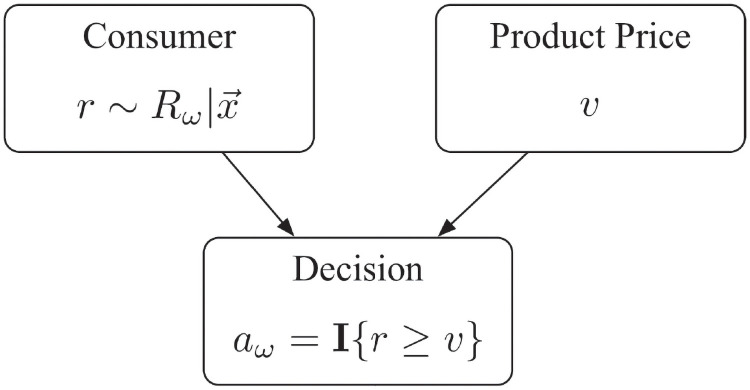
How consumers reach a purchasing decision when offered product *ω* at price *v*.

That is, we assume that after drawing an instant RP $r∼R_{\omega }|{\vec{x}}_{}$, the customer’s decision is determined by the relationship between *r* and *v* (see Fig 4). Then it is explicit that the relationship between the purchasing decision random variable $A_{\omega ,v}|{\vec{x}}_{}$ and the reservation price random variable $R_{\omega }|{\vec{x}}_{}$ is

$$\begin{array}{c} A_{\omega ,v}\;|\;{\vec{x}}_{}\;=\;I\left\{v\leq R_{\omega }|{\vec{x}}_{}\right\}. \end{array}$$ (3)

We can also derive the *purchasing probability function* $PPF_{\omega }\left({\vec{x}}_{},v\right)$, *i.e.*, the probability that consumer ${\vec{x}}_{}$ will buy product *ω* at price *v*, to be

$$\begin{array}{c} PPF_{\omega }\left({\vec{x}}_{},v\right)\;\overset{\Delta }{=}\;Pr\left(\;A_{\omega ,v}=1\;|\;{\vec{x}}_{}\;\right)\;=\;Pr\left(\;R_{\omega }\geq v\;|\;{\vec{x}}_{}\;\right)\;=\;1-F_{R_{\omega }|{\vec{x}}_{}}\left(v\right) \end{array}$$ (4)

where $F_{R_{\omega }|{\vec{x}}_{}}\left(\cdot \right)$ is the cumulative distribution function (CDF) of consumer ${\vec{x}}_{}’\text{s}$ RP for product *ω*.

Note that this decision-making process nicely matches the common understanding of RP–*i.e.*, the highest price a consumer is willing to pay for a unit of a certain product or service [12].

### 3.4 Data collection and format

As shown in the previous section, RPs and purchasing decisions are closely related to each other, which suggests that we can indirectly infer consumers’ RPs for a certain product from the purchasing decisions of these (and other) consumers. Therefore, for a certain product *ω*, instead of directly asking consumers for their instant RPs *r*_*i*_, we instead collect (non-)transaction data–*i.e.*, the decisions of many (earlier) consumers *a*_*i*,*ω*_ on whether they will purchase *ω* at various different prices *v*. Each observation in the dataset *D*_*ω*_ of product *ω* is a vector in the format (*x*_*i*_,*v*_*i*,*ω*_,*a*_*i*,*ω*_). An example dataset appears in Table 1.

**Table 1:** An example dataset of some product *ω*.

features of consumers ${\vec{x}}_{i}$				price *v*_*i*,*ω*_	decision *a*_*i*,*ω*_
age	gender	monthly income	…		
18	male	$200	…	$3.50	0
26	female	$3000	…	$5.00	1
…	…	…	…	…	…
28	female	$2000	…	$4.5	0

Traditional RP models require consumers to be highly involved in a sophisticated elicitation procedure in order to make them understand that telling the true RP is their optimal choice. However, our data collection process is significantly simpler and does not make assumptions about consumers’ understanding, as we do not ask consumers to report their RPs directly.

## 4 Reservation price prediction models

This section first summarizes the main ideas from survival analysis (including censoring), to show how we can use survival prediction techniques to learn the consumer-specific RP distribution from the (non-)transaction data. We then introduce three popular survival models and one recent effort from the machine learning community for predicting subject-specific survival distributions.

### 4.1 Relation to survival analysis

Typically survival analysis focuses on time-to-event data, where the variable of interest is the death/event time *T*. In general, survival models try to learn the survival function,

$$\begin{array}{c} {\hat{S}}_{T|{\vec{x}}_{}}\left(t\right)\;=\;1-F_{T|{\vec{x}}_{}}\left(t\right), \end{array}$$ (5)

from event and censored (left, right, or interval) data. This task differs from ordinary regression as it must deal with censored observations at training time, which are incomplete observations of the event time *T*.

**Fig 5:**
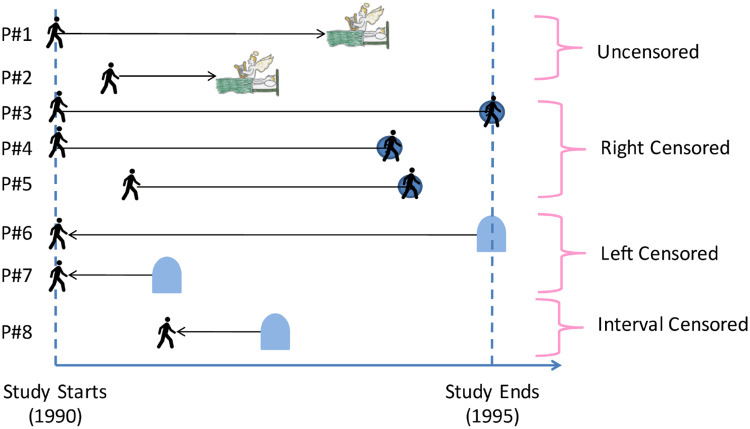
Uncensored observations. (where the patient’s actual time of death is observed), as well as right, left and interval censored events, where the time of death can only be bounded (providing a lower bound for right censored, and upper bounds for left censored, and an interval for interval censored). The “walking” figure shows the patient is alive at that time (the blue circle means this is the last time the patient was known to be alive), the angel emerging means that patient died at that time, and the tombstone symbol means the patient is known to be dead at that time (although s/he might have died earlier).

Fig 5 suggests a training sample, perhaps for a breast cancer study, where the variable of interest is the patient’s time of relapse. Here, we know the actual time when some patients relapse (P#1 and P#2). Some other patients may still be non-relapsed when the study ends (*e.g.*, P#3), and others may drop out of the study (P#4, P#5); here, we will never know her actual time of relapse *T*. All we know is that her relapse time is **after** her *censored time*–call it *c*_*r*_(*P*#*i*)–which is only partial information about *T*. This is called *right censoring*, as the unknown event time *T* is on the “right” side of the right-censored time, *c*_*r*_(*P*#*i*)–*i.e.*, *T* > *c*_*r*_(*P*#*i*). For yet other patients, the unknown true event time is **before** a certain time *c*_*l*_(*P*#*j*). For example, imagine finding that a patient gets her first examination six months after the study began, and is then diagnosed as having already experienced a relapse. In this case, all we know is that the relapse happened in the first six months. This is called *left censoring*; see P#6 and P#7 in Fig 5. Here, we only know that the subject had already relapsed at the measured time, but not when she had relapsed–*i.e.*, *T* < *c*_*r*_(*P*#*j*). Finally, we may sometimes know that the unknown true event time is **in** a certain time range [*t*_1_,*t*_2_]–*e.g.*, perhaps if patients take yearly examinations, then if a patient is diagnosed as having a relapse, then we only know that the relapse time is in the previous one year. See P#8.

Now observe that a consumer’s RP is similar to a patient’s survival time, in that the observations (purchasing transactions) provide only censored versions of the information needed. Consider a purchasing transaction–*e.g.*, when MrA bought a pen for $2. Here, we only know that MrA’s true RP for this pen is greater than or equal to $2. Similarly, if MsB chooses not to buy this pen for $5, this “non–purchasing transaction” means that MsB’s true RP is less than the $5. That is, for $r∼R_{\omega }\;|\;{\vec{x}}_{}$:

**Purchasing transaction:** *a*_*ω*_ = 1 ⇔ *r*_*ω*_ ≥ *v*
**Non-purchasing transaction:** *a*_*ω*_ = 0 ⇔ *r*_*ω*_ < *v*

If we take the RP *R* as the variable of interest, instead of time *T*, then the purchasing (resp., non-purchasing) observations in the RP setting are equivalent to right censored (resp., left censored) observations in the survival analysis setting, as each purchase means the price was a *lower bound* of the consumer’s true reservation price (just like the right-censored time is a *lower bound* of a patient’s time of death); and each non-purchase means the offered price is an *upper bound* of consumer’s true reservation price (just like the left-censored time exceeds the *upper bound* of a patient’s time of death). To be more clear, Table 2 shows the matching relationship between the terminologies in these two settings.

**Table 2:** Matching terminology and symbols.

Survival Analysis	Reservation Price
Event time *T*	Reservation price *R*
Survival distribution *f*(*t*)	RP distribution *f*(*v*)
Survival function *S*_*T*_(*t*)	Purchasing probability function *PPF*(*v*)
Left censored observation *c* = *l*	Non-purchasing transaction *a* = 0
Right censored observation *c* = *r*	Purchasing transaction *a* = 1
Uncensored observation *c* = *u*	Observation of true RP (not used in this model)

The final line illustrates one important difference between these models, in that most survival studies include complete observations about *some* subjects–*i.e.*, we know when some patients actually died. In our RP estimation task, however, we have no complete observations of consumers’ RPs at all, as every instance is either left- or right-censored. (Section 1.3 summarized other works that made similar connections).

**Fig 6:**
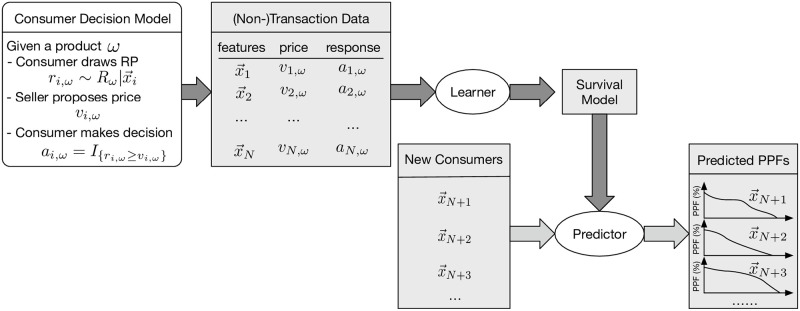
Illustration of the data-generation, learning process and how it works on new instances.

Nevertheless, this connection allows us to utilize survival models to learn the distribution of *R* using the (non-)purchasing transactions. Fig 6 illustrates the whole learning system and how it works on new consumers.

### 4.2 Survival models

This subsection introduces several (three classic, and one recent) survival models: Kaplan-Meier estimator [31], Cox proportional hazard model [32], accelerated failure time model [33], and the multi-task logistic regression (MTLR) model [34]. In the context of survival analysis, the Kaplan-Meier model estimates an entire population’s survival distribution whereas these other models estimate *individual survival distributions* (ISDs) [35], and hence we refer to these other models as ISD models.(Of course, these are only a few of the many different survival analysis models; we chose only these four models as they are standard, and/or representative of major classes we want to examine). We will test their performance in Section 6, to evaluate the effectiveness of our framework of RP.

#### 4.2.1 Kaplan-Meier estimator

The Kaplan-Meier (KM) estimator [31] is an empirical non-parametric model that estimates the survival function *S*(*t*), Eq 5. This tool is widely used in clinical studies for comparing the survival curves of two subpopulations in order to identify the risk factors–*i.e.*, the features important to survival.

For a dataset consisting of only uncensored and right censored data, the empirical estimate of *S*(*t*) is

$$\begin{array}{c} \hat{S}\left(t\right)=\prod_{j:\tau_{j}<t}(1-\frac{d_{j}}{r_{j}}), \end{array}$$ (6)

where *τ*_1_,*τ*_2_,…,*τ*_*K*_ are the set of all *K* distinct death times in the dataset, *d*_*j*_ is the number of deaths at time *τ*_*j*_, and *r*_*j*_ is the number of subjects at risk right before *τ*_*j*_ (*i.e.*, the number of subjects who died or were censored at or after *τ*_*j*_).

Since an RP dataset only consists of left- and right-censored data, but no event data, we have to resort to the Expectation-Maximization approach of Turnbull [36] to estimate the survival curve, where both left and right censored data are treated as interval censored data.

Notice that the KM estimator does not consider the features of the subjects, which means it predicts the same survival curve for all subjects and thus is not personalized. We still include it here for completeness, as it is one of the most widely accepted and used models in survival analysis. We implemented this using the ic_np function in the R package icenReg [37] for the Turnball estimator.

#### 4.2.2 Cox proportional hazards model

The Cox proportional hazards (Cox) model is a semi-parametric model designed for comparing the survival time of two populations or to identify the risk factors critical to survival [32]. Unlike the KM model, the Cox model uses the subject’s features and works with the hazard function(The hazard function is also called the “failure rate” since it reflects the subject’s instantaneous rate of failure).

$$\begin{array}{c} \lambda \left(t\right)\;=\;\lim_{\Delta t\to 0}\frac{Pr(\;t\leq T<t+\Delta t\;|\;T\geq t)}{\Delta t}\;=\;\frac{f(t)}{S(t)},\; \end{array}$$ (7)

instead of the survival function, where *f*(*t*) = *Pr*(*T* = *t*) is the probability that the patient dies at time *t*.

The Cox model models the hazard function as

$$\begin{array}{c} \lambda \left(\;t\;|\;x_{i}\right)=\lambda_{0}\left(t\right)\;\exp\left({\vec{x}}_{i}^{T}\theta \right), \end{array}$$ (8)

where *λ*_0_(*t*) is the baseline hazard function, and *θ* is learned from a data sample. Here, as the relative influence of each feature *x*_*i*,*k*_ depends linearly (“proportionally”) on the corresponding coefficient *θ*_*k*_ (albeit in the exponent), this is called a *proportional hazard model*.

**Fig 7:**
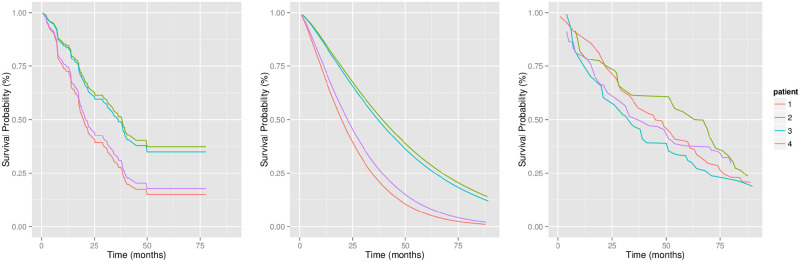
Survival curves for several patients from North Alberta Cancer Dataset [34], generated by (a) Cox model; (b) AFT model; (c) MTLR.

One of the advantages of this model is that we can estimate *θ* by maximum partial likelihood estimation [32], which requires no knowledge of the baseline hazard *λ*_0_(*t*). This simplifies the task of identifying the risk factors. However when it comes to the prediction task, the proportional hazard assumption restricts the shapes of predicted survival curves of all patients to be essentially the same, as shown in Fig 7(a). This means its predictions on subjects’ survival rates might not be calibrated [34].

Similar to our analysis of Kaplan-Meier model (Subsection 4.2.1), we again treat both left and right censored data as interval censored data and utilize the Cox model designed for interval censored data to estimate our model [38, 39]. Specifically, we use the icenReg [37] package again but use the ic_sp function.

#### 4.2.3 Accelerated failure time model (Tobit model)

The accelerated failure time model (AFT) is a parametric model that directly models the distribution of *T* with some parametric distribution [33], as shown below:

$$\begin{array}{c} \log T_{i}=\theta^{T}{\vec{x}}_{i}\;+\;\delta \;\epsilon , \end{array}$$ (9)

where *δ* is the scale parameter and *ϵ* is the error term. Different distributions of *ϵ* yield different forms of the AFT model. The commonly used distributions for *ϵ* include the Weibull distribution, log-logistic distribution, and the log-normal distribution, and the normal distribution. With ∊∼N(0,1) (Gaussian distribution with zero mean and unit variance), the AFT model is actually equivalent to the well-known Tobit model in the economics literature [40].

In the AFT model, the effect of covariates is to accelerate/decelerate the scale of life time, while in the Cox model, the effect of covariates is to multiply the hazard by a constant. Fig 7(b) shows an example of the predicted survival curves of four patients in a survival dataset, by the AFT model with *ϵ* following the log-normal distribution. In our experiments, we fit the AFT model with *ϵ* following the log-normal distribution using the function survreg in the R package survival [41].

#### 4.2.4 Multi-task logistic regression

Multi-task logistic regression (MTLR) is a recent effort from the machine learning community to produce a patient-specific survival function, which works well, according to several criteria [34]. Unlike the earlier models, MTLR does not make any explicit assumptions nor restrictions about the hazard function nor the shape of survival curves, meaning the MTLR survival curves of different individuals can be very different and can intersect with each other. This offers greater prediction capacity and flexibility. Fig 7(c) shows the predicted survival functions from MTLR for four patients [34].

MTLR first discretizes the continuous time axis into *K*+1 time points {*τ*_0_,*τ*_1_,*τ*_2_,…,*τ*_*K*_}, with *τ*_0_ = 0 and *τ*_*K*_ = ∞, and then transforms the survival function prediction task into a sequence of binary probabilistic classification tasks, by constructing initially a logistic regression model for each time point *τ*_*j*_,*j* = 1,…,*K* − 1:

$$\begin{array}{c} Pr\left(\;y_{j}=0\;|\;{\vec{x}}_{}\;\right)={\left(1+\exp({\vec{x}}_{}^{T}\cdot \vec{\theta_{j}}+b_{j})\right)}^{-1} \end{array}$$ (10)

where ${\vec{\theta }}_{j}$ and *b*_*j*_ are the parameters associated with the *j*^*th*^ time point and *y*_*j*_ = **I**{*T* < *τ*_*j*_} indicates if the subject ${\vec{x}}_{}$ has incurred an event before *τ*_*j*_. (We earlier explored various rules for setting *K*, and found that $K\approx \sqrt{N}$, where *N* = *N*_*e*_+*N*_*c*_ is the total number of individuals in the study, works effectively).

Then if we (for now) treat the classifiers as independent, we have the probability mass function (PMF) of $\vec{y}$ as

$$\begin{array}{c} \tilde{Pr}\left(\;\vec{y}\;|\;{\vec{x}}_{}\right)=\frac{\exp(\sum_{k=1}^{K-1}\left({\vec{x}}_{}^{T}\cdot \vec{\theta_{k}}+b_{k}\right)y_{k})}{\prod_{k=1}^{K-1}\left(1+\exp\left({\vec{x}}_{}^{T}\cdot \vec{\theta_{k}}+b_{k}\right)\right)} \end{array}$$ (11)

However, as we must prevent the case that *y*_*j*_ = 1 and *y*_*j*+1_ = 0 from holding (that is, after someone dies, that person cannot come back alive–the “No Zombie” rule), the normalization term is the summation of the unnormalized “probability” of these *K* legal $\vec{y}\text{s}$, which are (1, 1,…,1, 1), (0, 1,…,1, 1),…, (0, 0,…,0, 1), and (0, 0,…,0, 0). The final form of the PMF (probability mass function) of *T* is

$$\begin{array}{c} \begin{array}{cc} & Pr(\;\tau_{j-1}\leq T<\tau_{j}\;|\;{\vec{x}}_{}\;)\\ & =\;\tilde{Pr}\left(\;\vec{y}\;=\;\left(y_{1}=0,\;\ldots ,\;y_{j-1}=0,\;y_{j}=1,\;\ldots ,\;y_{K-1}=1\right)\;|\;{\vec{x}}_{}\right)\\ & =\;\frac{\exp(\sum_{k=1}^{K-1}y_{k}\;\left({\vec{x}}_{}^{T}\cdot \vec{\theta_{k}}+b_{k}\right))}{Z(\Theta ,B,{\vec{x}}_{})}\;=\;\frac{\exp(\sum_{k=j}^{K-1}\left({\vec{x}}_{}^{T}\cdot \vec{\theta_{k}}+b_{k}\right))}{Z(\Theta ,B,{\vec{x}}_{})}, \end{array} \end{array}$$ (12)

where $\Theta =({\vec{\theta }}_{1},{\vec{\theta }}_{2},...,{\vec{\theta }}_{K-1})$, *B* = (*b*_1_,*b*_2_…*b*_*K*−1_) and

$$\begin{array}{c} Z\left(\Theta ,B,{\vec{x}}_{}\right)\;=\;\sum_{j=1}^{K}\exp(\sum_{k=j}^{K-1}\left({\vec{x}}_{i}^{T}\cdot \vec{\theta_{k}}+b_{k}\right)) \end{array}$$

is the normalization term.

Then one can derive the log-likelihood function of a dataset $D=\{\left[\vec{x_{i}},t_{i}\right]\}$, where the first *N*_*e*_ instances are uncensored and the remaining *N*_*c*_ are right and/or left censored:

$$\begin{array}{ccc} LL(\;D;\;[\Theta ,B]\;) & = & \sum_{i=1}^{N_{e}}\sum_{k=1}^{K-1}\left({\vec{x}}_{i}^{T}\cdot \vec{\theta_{k}}+b_{k}\right)y_{k}\left(t_{i}\right))\\ & & +\;\sum_{i=N_{e}+1}^{N_{e}+N_{c}}\log[\sum_{j=1}^{K}c_{j}\left(t_{i}\right)\exp(\sum_{k=j}^{K-1}\left({\vec{x}}_{i}^{T}\cdot \vec{\theta_{k}}+b_{k}\right))]\\ & & -\;\sum_{i=1}^{N_{e}+N_{c}}\log(Z(\Theta ,B,{\vec{x}}_{i})), \end{array}$$

where *y*_*k*_(*t*_*i*_) = **I**{*t*_*i*_ < *τ*_*k*_}, and also *c*_*j*_(*t*_*i*_) = **I**{*t*_*i*_ < *τ*_*j*_} for right censored observations and *c*_*j*_(*t*_*i*_) = **I**{*t*_*i*_ ≥ *τ*_*j*−1_} for left censored observations.

MTLR is accessible as a web-tool (http://pssp.srv.ualberta.ca/) but our experiments use the R implementation provided in the MTLR [42] package.

## 5 Dataset

### 5.1 Data collection

While there are many datasets of financial transactions, essentially all report only the actual purchases, but not the “non-purchases”—*i.e.*, they do not report situations where a consumer has *declined* an offer. For our stochastic RP setting, we need a dataset that contains both purchases and non-purchases. (While the donation dataset used in KDD Cup 1998 [43] does provide “non-donate” transactions, these non-donations only happen when a donor’s “reservation donation” is zero, which means that this dataset provides no meaningful left-censored observations).(While relatively few datasets record such non-transactions, they are clearly available, as many websites routinely collect this information about the users for future marketing advertisements [44, 45]. We wonder if many existing datasets do not collect is just because none of the standard analyses have demonstrated a benefit to this information. Perhaps the results of this research will motivate future researchers to collect this important information).

We therefore designed and executed our own online survey on Qualtrics, asking subjects from Amazon Mechanical Turk to provide information about themselves, and about their interest in purchasing each of four different specific-types of chocolate bar.

Here, we acquire one datasetand use a one-hot encoded feature to identify the brand of chocolate. For each consumer, we collected 41 features, *e.g.*, the consumer’s demographics information, and preference towards the chocolate brand and flavor, the time when s/he ate her/his last meal and so on. Note that the subjects did not purchase any product in the survey; they just provided information, for which they were paid. (Note (1) we obtained the appropriate ethics permission for this study with human participants; (2) the dataset is publicly available at https://github.com/haiderstats/reservation-prices; and (3) more details about the survey appear in Appendix A in S1 File, or visit https://qtrial2014.az1.qualtrics.com/SE/?SID=SV_0kycgJjTgOj5Z8p).

The purpose of this survey is to collect relevant information, to help us in evaluating our various models. Therefore, we directly ask each participant *i* to provide his/her instant RP *r*_*i*,*ω*_ for the product *ω*; we then used this to determine their responses *a*_*i*,*ω*_ to certain offers, *i.e.*, (non-)transaction data, following the decision-making process proposed in Subsection 3.2. *N.b.*, our *learning* algorithms do NOT use those *r*_*i*,*ω*_’s–instead, they just use the (non-)transaction data; see Fig 6. We only used the collected *r*_*i*,*ω*_ values as a way to evaluate our learners.

To explore the utility of the features (the Survey Questions; see Appendix A in S1 File) for our RP estimation task, we ran a simple Cox “univariate feature selection” process on this data, to identify which individual features are “relevant”, at *p* < 0.05; see Table 3. Note however that none of the learning algorithms used that data.

**Table 3:** Features that Cox-feature-selection considered relevant, at *p* < 0.05.

Id	Question
-	Brand of Chocolate
A.3.9	How likely will you recommend this chocolate to your friends?
A.3.10	How tasty do you believe the chocolate is?
A.4.3.e	What kind of features of chocolate do you like—fruit flavor?
A.4.4	On average, how much do you pay on chocolate in each grocery shopping?
A.4.5	For all the possible chocolates available (weight: 100g), what is the highest price you are willing to pay?
A.4.6.e	When making chocolate purchase decisions, how important is the shape & looking of chocolate on affecting your decision?
A.5.4	What is your employment status?

### 5.2 Data quality

To ensure that our data quality is good and the reported RPs are accurate, our online survey included five attention-check questions, one RP understanding question and a two-step RP elicitation procedure [46]. We eliminated any subject who failed any attention-check or RP understanding question or who showed any inconsistency in his/her answers about RP. We also eliminated blatantly ridiculous responses—*e.g.*, a subject willing to pay $10000 for a 100g chocolate bar.

This left 722 responses (out of 1080 submissions) qualified for each of the four chocolates, leading to an overall dataset size of 722 × 4 = 2888 instances, with 41 features describing consumer preferences/demographics and a one-hot encoded feature identifying the chocolate brand. Table 4 reports the median, mean (and standard deviation) of the consumer’s reported RPs.

**Table 4:** Median, mean (+ std) RP for the chocolate in the four datasets, over the 722 consumers; also retail price for each.

	Lindt	Godiva	Valrhona	Hersheys
Median of RP (*s*_*ω*_)	4.50	3.99	2.99	1.25
Mean of RP	3.88	4.84	2.94	1.48
Std of RP	1.89	2.92	2.08	1.05
Retail Price	6.00	10.00	7.50	2.00

While we tried to produce a dataset with good quality, the hypothetical response bias [25] cannot be completely avoided, as there were no real purchases. Fortunately, as our goal is to evaluate the performance of survival models within our novel framework of RP, this systematic bias will not be a serious issue. When online retailers later collect (non-)transaction data in practice, the consumers will be making purchases, which will mitigate this hypothetical response bias.

### 5.3 Generation of (non-)transaction data

After acquiring the true RPs and features of the consumers, we simulated a (non-)transaction data collection session by first sampling one query price *v*_*i*,*ω*_ for each consumer from a stretched Chi-Square distribution–*i.e.*, for each dataset, we first we set the parameter *k* in *χ*_*k*_ to be the mean of the RPs, and then used a linear mapping to match the variance of the distribution $\chi_{k}^{2}$ with the variance of the RPs. We then determined the consumer’s response *a*_*i*,*ω*_ following the decision-making process defined in Subsection 3.2–*i.e.*, the consumer’s purchasing decision is simply

$$\begin{array}{c} a_{i,\omega }=\{\begin{array}{cc} \text{1}\;\text{[yes]} & \text{if}\;v_{i,\omega }\leq r_{i,\omega }\\ \text{0}\;\text{[no]} & \text{otherwise} \end{array} \end{array}$$ (13)

This led to a dataset where each row is described by 41 features, the brand indicator, and also an offer price, *v*_*i*,*ω*_ and response bit, *a*_*i*,*ω*_, for each *i*^*th*^ consumer for the *ω*^*th*^ brand. That is, the format of this dataset strictly conforms with the example dataset shown in Table 1 (see also Fig 6).

**Fig 8:**
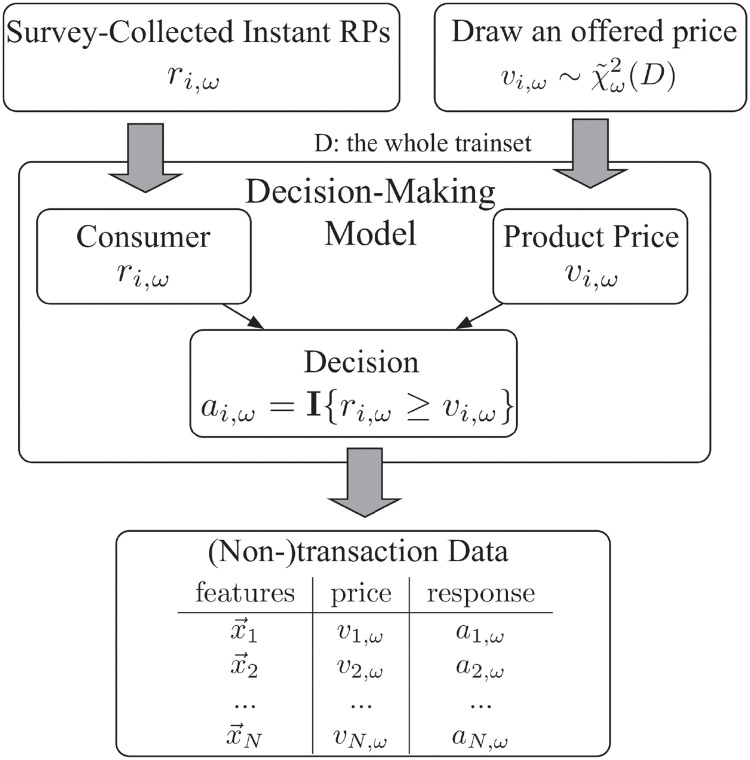
Procedure of generating (non-)transaction data.

Note in particular that the dataset does *not* include the consumer’s RP *r*_*i*,*ω*_ nor did we use the true RP data in training nor in the hyper-parameter selection via cross validation. The true RP data is only available in the final testing phase for evaluating the RP prediction performance. The whole procedure is outlined in Fig 8.

## 6 Experimental results

While Concordance (aka C-index) is a fairly standard measure for evaluating survival models [13], this section presents three other measures that are more relevant for this marketing task–including expected profit. All results are based on ten-times repeated ten-fold cross-validation where, for each of our four survival models (KM, Cox, AFT, and MTLR), we train a model on 9/10 of the subjects; we then use that learned model to produce a “RP distribution” for each of the remaining 1/10 of the subjects—we then repeat this process ten times. With the exception of KM, these models train on one dataset containing all four types of chocolates. Since KM is a population-based model we build four separate KM models–one for each type of chocolate. For MTLR, within each fold we execute an internal three-fold cross validation to select the best hyper-parameter, *e.g.*, regularization constant. All significance tests presented use the two-sided *t*-test statistic given by Bouckaert and Frank [47], which corrects for the high Type II error and low replicability of significance tests involving cross-validation. For transparency all experimental code and data are publicly available (https://github.com/haiderstats/reservation-prices).

### 6.1 Mean absolute error

Given the learned CDF ${\hat{F}}_{R_{\omega }|{\vec{x}}_{i}}\left(v\right)$ of consumer ${\vec{x}}_{i}$’s RP for product *ω*, we use the median RP as the prediction for consumer ${\vec{x}}_{i}$’s RP value:

$$\begin{array}{c} \text{Median}\left(\;R_{\omega }\;|\;{\vec{x}}_{i}\;\right)\;=\;{\hat{r}}_{i,\omega }\;s.t.{\hat{F}}_{R_{\omega }|{\vec{x}}_{i}}\left({\hat{r}}_{i,\omega }\right)\;=\;0.5. \end{array}$$ (14)

(We use the *median* price point as the RP prediction, as it is more robust than *mean*). As we have collected the consumers’ true instant RP *r*_*i*,*ω*_, we can compute the mean absolute error (MAE) of our predicted RPs,

$$\begin{array}{c} MAE_{\omega }=\frac{1}{N}\sum_{i=1}^{N}\left|{\hat{r}}_{i,\omega }-r_{i,\omega }\right|, \end{array}$$ (15)

where *N* is the number of consumers. Note that we cannot use this criterion in internal cross-validation to select hyper-parameters, because the learners do not have access to the true RP.

**Fig 9:**
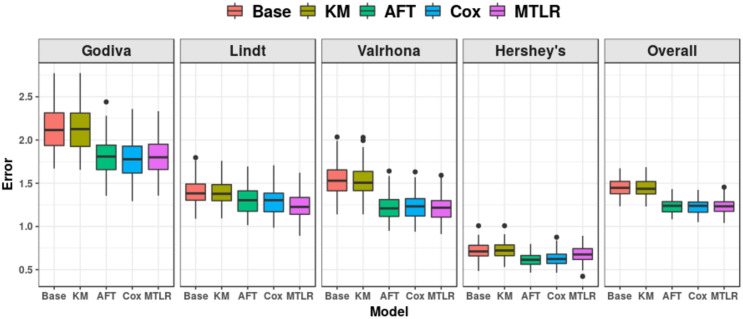
Mean absolute error (MAE) over ten-times repeated ten-fold CV.

Fig 9 (and Table 6 in Appendix B of S1 File) shows the ten-times repeated ten-fold cross validation MAE for KM and the 3 ISD models, as well as a “cheating” baseline model. This “cheating” baseline utilizes the consumers’ true RPs, which are not available to the learners, and computes the median value of RP in the training set to use as its prediction on the test set. We also include the “Base” model, which is the best “single price” (not personalized) model possible, given the reservation price information: Here, we first compute the true RPs of the consumers in the training set then use its median value as the offer price to each consumer in the test set. (This is oracle-based as the learners do not know the consumers’ true RPs).

Despite this, we still found that the ISD models (AFT, Cox, MTLR) achieve better (that is, lower) MAE than the cheating baseline and the KM model, across all brands of chocolate. Specifically, for the overall average error (far right subfigure of Fig 9), two-sided *t*-tests show the performance of all ISD models were significantly better than KM and the baseline, *p* < 0.001, but no ISD model was significantly better than another. (While the “cheating model” did know a lot about the current consumer, it did not use information about the other consumers; our results show that models learned from just the “legal” information about those other consumers, can do better that ones based on “illegal” information about the current consumer).

### 6.2 Binary classification accuracy

This evaluation criterion tests if the learned models can accurately predict the consumer’s response to our offer of *ω* at price *v*. This too is very important in real applications. Here, each predictor predicts the response using

$$\begin{array}{c} {\hat{a}}_{i,\omega }=\{\begin{array}{cc} \text{yes} & \text{if}\;PPF_{\omega }\left({\vec{x}}_{i},v\right)=1-{\hat{F}}_{R_{\omega }|{\vec{x}}_{i}}\left(v\right)\;\geq \;0.5\\ \text{no} & \text{otherwise} \end{array} \end{array}$$ (16)

We then compute the classification accuracy as

$$\begin{array}{c} ACC\left(\omega \right)=\frac{1}{N}\sum_{i=1}^{N}I\left\{{\hat{a}}_{i,\omega }=a_{i,\omega }\right\}. \end{array}$$ (17)

**Fig 10:**
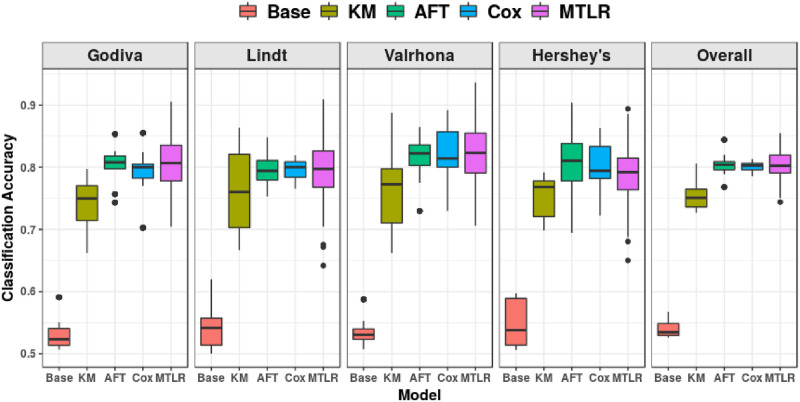
Ten-times repeated ten-fold cross validation classification accuracy, for each chocolate individually, and overall.

Fig 10 shows that the classification accuracies of all four survival models (even the non-personalized KM) are significantly better (*p* < 0.001) than the “random guess” baseline, *i.e.*,

$$\begin{array}{c} \text{baseline}\;=\;\frac{\max\{\#\text{purchasing}\_\text{transactions},\#\text{non}-\text{purchasing}\_\text{transactions}\;\}}{\#\text{all}\_\text{transactions}} \end{array}$$

Additionally, in overall accuracy (far right subfigure of Fig 10), all ISDs outperformed KM, all *p* < 0.001, but did not significantly outperform one another. Table 7 in Appendix B of S1 File, provides the detailed information.

### 6.3 Profit using a simple pricing strategy

This section explores whether survival models within our RP framework can lead to real profit in practice. We use a pricing strategy that is simple and intuitive, which aims to maximize the expected profit and relies on good estimates of the PPF.

As we have a predicted purchasing probability function $\hat{PPF_{\omega }}\left({\vec{x}}_{i},v\right)=1-{\hat{F}}_{R_{\omega }|{\vec{x}}_{i}}\left(v\right)$ for each consumer ${\vec{x}}_{i}$, we know the predicted expected profit by offering *ω* at price *v* to ${\vec{x}}_{i}$ would be $\left(v-c\right)\cdot {\hat{PPF}}_{\omega }\left({\vec{x}}_{i},v\right)$ where *c* is the seller’s cost to produce *ω*. Here, the seller should therefore offer the product *ω* to ${\vec{x}}_{i}$ at the price ${\hat{v}}_{i}\left(c\right)$ with maximum expected profit:

$$\begin{array}{c} {\hat{v}}_{i}\left(\;c\;\right)=\underset{v}{arg max}\;\left\{\;\left(v-c\right)\cdot {\hat{\;PPF}}_{\omega }\left({\vec{x}}_{i},v\right)\;\right\}\;. \end{array}$$ (18)

The true mean profit *PFT*_*ω*_(*c*) for product *ω*, with production cost *c*, is

$$\begin{array}{c} PFT_{\omega }\left(c\right)=\frac{1}{N}\sum_{i=1}^{N}I\left\{{\hat{v}}_{i}\left(c\right)\leq r_{i,\omega }\right\}\cdot \left({\hat{v}}_{i}\left(c\right)-c\right). \end{array}$$ (19)

Unfortunately, due the price variability among retailers, there is no single retail price of the chocolates; moreover, we realized that the consumers in our population probably had limited interaction with the products–perhaps only through this survey. This motivated us to set the retail price *s*_*ω*_ of each chocolate bar *ω* to be its median reservation price, over the consumers–see Table 4.

Additionally, we also do not know the production costs. We therefore considered a range of possible production costs, at different proportions of the retail price *c*_*ω*_∈ {0.10*s*_*ω*_,0.15*s*_*ω*_,…,0.85*s*_*ω*_,0.90*s*_*ω*_}. Note that Gilbert [48] claims that the most likely cost, for chocolate, is ≈0.75*s*_*ω*_, based on the retail costs and margin accounting for 28% of the retail cost of chocolate.

Below we consider 4 different ways to determine how much the seller should charge for this product, ${\hat{v}}_{i}\left(c\right)$; see Table 5. So far, we have considered survival-based methods, including some that are personalized (ISD models: AFT, Cox, MTLR), and one that is not (KM). We also consider using the retail price (median reservation price *s*_*ω*_) as a fixed price used for all customers.

**Table 5:** Different approaches to set prices.

	Personalized	Not Personalized
Survival-based	AFT, Cox, MTLR	KM
Not Survival-based	LDA, LR, NB	Retail Price

For comparison, we also evaluate the performance of three typical machine learning (ML) based probabilistic classifiers–*viz.*, naïve Bayes (NB), logistic regression (LR) and linear discriminant analysis (LDA)–on the profit criterion, as these models can also be used to estimate PPF, though in a different manner: Here, for each product *ω*, we define the purchasing decision variable as *A* ∈ {0,1} and use $\left({\vec{X}}_{},V\right)$ as the input variables, where *V* is the product price variable. That is, for each ρ∈{NB,LR,LDA}, the learned *PPF*^*ρ*^ model corresponds to

$$\begin{array}{c} PPF^{\rho }\left({\vec{x}}_{i},v\right)\;quad=\;Pr_{\rho }\left(\;A=1\;|\;{\vec{X}}_{}={\vec{x}}_{i},\;V=v\;\right) \end{array}$$

(To simplify notation, we do not include the *ω*).

Note the probability that a consumer will purchase a product should be *monotonic* in the offer price–*i.e.*, if there is a 10% chance that a consumer will purchase an item if it is offered at $1, it should not be 50% at $2. We intentionally did not consider any nonlinear models as we found that they did not always exhibit this required property.

**Fig 11:**
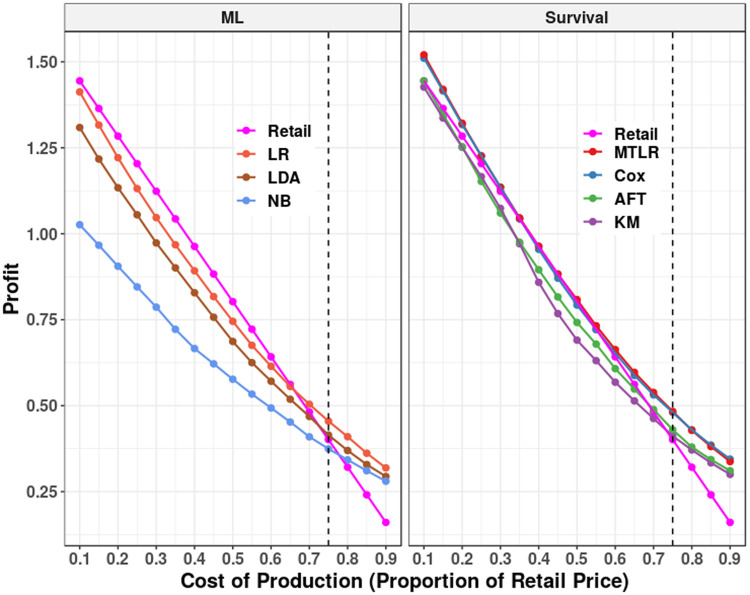
Overall average profit 10-times 10-fold cross validation results. On left, the average profit generated by the ML models– *i.e.*, LR, LDA, and NB. On right, the average profit for the survival models–*i.e.*, KM, AFT, Cox, and MTLR. The retail price model (in pink) is included in both sections for reference. The vertical dashed line is placed at the most likely real proportion of cost (0.75). See details in Table 8 in S1 File.

For each type of chocolate, for each of these 3 + 1 + 3 + 1 = 8 methods (Table 5), we computed the (10-times 10-fold CV) average profit for each of the 17 proposed costs *c*_*ω*_. Fig 11 summarizes these average profit across all brands of chocolate, both by survival models (on right) versus ML models (on left), where each plot also includes the retail price model (the profit associated with selling the product at the standard price–see Table 4–in pink) as a reference. For completeness, this profit, for each chocolate brand individually, is shown in Figs 12–15 in Appendix B of S1 File.

For the ML models, LR is strictly better than both the LDA and NB models, but still worse than the retail price model for *c*_*ω*_ ∈ [0.10,0.65]*s*_*ω*_. Similarly, both AFT and KM are worse than using the retail price for *c*_*ω*_ < 0.70*s*_*ω*_. The Cox and MTLR models either match or are superior to the retail price model–Cox and MTLR make a significantly higher profit (*p* < 0.05) than the retail model at majority of costs–all except *c*_*ω*_ ∈ [0.30,0.55]*s*_*ω*_. Cox and MTLR’s profits do not significantly differ from each other at any cost but are significantly higher than AFT and LR at all costs (*p* < 0.01). This result strongly supports our idea of estimating RP on the individual level and reinforces the approach of using survival models for such estimation.

### 6.4 Discussion

First, we saw that ISD models (AFT, Cox, MTLR), with no knowledge of the true RP, can beat the cheating baseline on the MAE evaluation criterion. This shows that even without direct measurement of consumers’ true RPs, but only censored observations (of other consumers), one can still produce pretty good estimates of a consumer’s RP. It suggests that our way of collecting data may work in practice for the RP estimation task.

Second, on all three evaluation criteria, the performance of the ISD models are generally much better than the non-personalized one, *i.e.*, KM. This is strong empirical support for modeling *consumer-specific RPs*, rather than a uniform RP. Moreover, the fact that the personalized MTLR and Cox models obtained significantly more profit than the retail price at a large majority of cost points, further bolsters this personalized approach.

Third, the strong performance of the MTLR and Cox models (both much better than the baselines) with respect to the *ACC* and *PFT* evaluation criteria, suggests that our way of estimating RP can be helpful in the real-world challenge of predicting if consumers would accept an offer or not, which will lead to higher profits. This is extremely useful for online retailers who want to conduct private promotions or general first degree price discrimination [49].

Note that we were initially surprised that our results were so good–indeed, apparently better than companies, who certainly must have seriously explored this pricing issue. We attribute our success to two factors: First, we were able to offer different prices to different consumers, which is probably not an option for products like chocolate bars. Second, we also had access to a number of characteristics for each consumer, which certainly helped our predictor; this might not always be available. We view our analysis as a proof-of-concept, to further demonstrate that it is possible to improve profit by offering personalized prices, assuming certain consumer features are known.

We also found that MTLR and Cox did extremely well—better than the other survival models considered—on maximizing profits. This suggests that MTLR and Cox are likely good choices for predicting RP distribution predictions, in general. Besides right censored and event data, MTLR can also handle left censored and interval censored data without modifications, while most packages of KM, AFT, and Cox only deal with event and right censored data. For this same reason, many other recent survival models (such as Random Survival Forests [50] and many recent deep learning models [16, 51–54]) cannot be applied without major modification.

The capability to more accurately infer how much specific consumers might be willing to pay for a particular product–*i.e.*, their individual reservation prices—is undoubtedly beneficial to sellers. However, converting this informational advantage into economic value for a firm is not a straightforward endeavor. In many settings, (near) perfect price discrimination–where the seller attempts to offer the product to each consumer at his/her exact reservation price (or just below it), as long as it exceeds the incremental cost of production–is not feasible due to the firm’s limited ability to make an individual consumer a strictly “private” price offer and prevent consumers from sharing price information among each other. Moreover, consumers may have a choice among competing offers by multiple sellers, and thus concurrently available offers must be incorporated along with individuals’ reservation prices. In addition, a person’s reservation price for a specific product may vary over time (*e.g.*, as a function of other purchase or consumption decisions), presenting yet another challenge for sellers who intend to use inferred consumer-specific reservation prices to guide the implementation of personalized pricing.

Finally, the prospect of using individual reservation prices—accurately inferred based on prior behavior—to generate personalized price offers has important ethical implications. Is it socially acceptable for a seller to charge different consumers a different price for the same product? On one hand, one could argue that such a practice might be unfair to consumers. On the other hand, though, the are many instances where some form of price discrimination is occurring in the marketplace (*e.g.*, based on when, where, or what quantity consumers buy, their prior purchases/loyalty, or their demographic characteristics), evidently without significant consumer backlash. It is difficult to predict how marketplace norms, and in particular consumers’ views towards sellers engaging in massively personalized pricing, might evolve. As a final observation on this issue, note that personalized pricing might actually *enhance* social welfare by providing a mechanism for serving some (*e.g.*, economically underprivileged) consumers who would not be able to afford a product if the seller (was required to) set a single, uniform price, by offering it to these individuals at prices that they can afford.

## 7 Future work

### 7.1 Transaction-specific RP estimation

We anticipate it would be straightforward to integrate the product features $\vec{Y}$ in the dataset, allowing us to estimate *(consumer, product)-specific* reservation prices–*i.e.*, estimating $R|{\vec{x}}_{},\vec{y}$ instead of $R|{\vec{x}}_{}$. As $R|{\vec{x}}_{},\vec{y_{1}}$ may be related to $R|{\vec{x}}_{},\vec{y_{2}}$, this might allow us to transfer the knowledge of RP between similar products, possibility allowing us to estimate a consumer’s RP for a new product, as long as we know the product features. However, as we currently only have have data about four similar products, it is not realistic for us to experiment on this task.

A more ambitious goal is to include other information, such as transaction time and transaction location. Our framework can easily model this case, as well.

### 7.2 Relevant behavioral indicators

We can consider including other relevant behavioral indicators in the model estimation. A consumer’s decision process involves spending time inspecting various offers provided by multiple sellers, whether s/he considers a particular option as a viable candidate (*e.g.*, adding an option to the shopping cart or wish list), or whether s/he revisits certain preferred options. The combination of certain behavioral features can help predict reservation prices that are particularly profitable for a specific segment of consumers. Online retailers, such as Amazon and Alibaba, follow similar practices by using targeted ads towards consumers who have inspected similar items. Nevertheless, such pricing strategies require companies to have significant resources for exploiting such behavioral indicators and assumes consumers have limited information about competing products offered by other companies.

### 7.3 Unbalanced data

In our study, we set the retail price to be the median reservation price, implying that half of consumers would purchase the product at the retail price. In real settings, most consumers will turn down most products—that is, most consumers will not accept most offers from online retailers. This means that most datasets will be (seriously) unbalanced [55], where the degree of unbalance will depend on several factors, such as the promotion strategy, distribution of offer price and the product itself. We plan to further study this direction, to see if survival models can handle such very unbalanced datasets.

### 7.4 Online predictor

Suppose we have two consumers ${\vec{x}}_{i}$ and ${\vec{x}}_{j}$ where ${\vec{x}}_{i}$ and ${\vec{x}}_{j}$ are very similar, then we find that ${\vec{x}}_{i}$ declines our offer for *ω* at *v*_*i*_ = $5. Should we then offer *ω* to ${\vec{x}}_{j}$ at a price higher than $5? Probably not, as ${\vec{x}}_{i}$ and ${\vec{x}}_{j}$ are similar. This example argues that we should generate the offers sequentially, utilizing the previous responses, as this may be better than generating the offers {*v*_*i*_} in a batch mode. This leads to many interesting contextual bandit issues, and associated analyses [56]. We plan to extend our system to this on-line context.

## 8 Contributions

Motivated by the new demands of e-commerce, we propose a novel framework of estimating consumer-specific reservation price, which consists of a consumer decision-making model, and a corresponding data collection method.

This framework has three major advantages over the traditional elicitation methods in the marketing literature, which help it meet the new demands of the e-commerce scenario:

- It captures the inherent uncertainty of reservation price, consistent with the discussion in Talluri and Van Ryzin [12], etc.
- It connects the RP estimation task to survival prediction, which allows us to use survival models (standard and novel) to perform an individual-level RP prediction based on consumer-specific information.
- It is much easier and more practical for on-line retailers to implement our framework than the traditional elicitation method, as our data collection method does not ask consumers to report their true RPs, but indirectly infer a consumer’s RP based on historical (non-)transaction data of other consumers.

The experimental results show that survival prediction models, especially Cox and MTLR, perform well on this task under three different criteria. This empirically suggests that our framework of learning an RP prediction model is meaningful and could be very useful in practice. Given this success based on a relatively small dataset, we anticipate that others may try this approach on larger datasets, with even greater success.

## Supporting information

S1 File (PDF)
