# Supplementary material for: Using survival prediction techniques to learn consumer-specific reservation price distributions
Source: PLoS One. 2021 Apr 29;16(4):e0249182. doi: 10.1371/journal.pone.0249182 (PMC8084175; doi:10.1371/journal.pone.0249182)
Supplement: S1 File — (PDF) [file pone.0249182.s001.pdf]

A Survey Questions

A.1 Verification of Respondents' Understanding of RP

1. Please read the explanation below about Reservation Price carefully. There are two follow-up questions that test your understanding of reservation price. If you fail to answer the test of understanding questions correctly three times, you will not be allowed to keep on participating in this study, and you will not get any payments.

   Review on Reservation Price:
   Definition: Your current reservation price is the maximum price you are willing to pay (WTP) for a certain unit of product or service right now. For a unit of product or service, if its price is equal to or lower than your current reservation price, you will buy it now; if its price is higher than your current reservation price, you will not buy it.

   Example: Supposed that Jason's current reservation price for a bottle of coke is $2.50, it means that AT THIS CURRENT MOMENT,
   If we offer the coke to Jason at price equal to or lower than $2.50 now, Jason will buy it.

   If we offer the coke to Jason at price higher than $2.50 now, Jason will not buy it.

2. Suppose that Tom's reservation price for a pen right now is (a random number), if we offer it to Tom at price (a random number), will he buy it or not?

   (a) Yes, Tom will buy it.

   (b) No, Tom will not buy it.

   (c) Not sure, Tom may buy it or not.

3. When will one choose to purchase a product?

   (a) the price of the product is equal or higher than his/her reservation price

   (b) the price of the product is equal or lower than his/her reservation price

   (c) the price of the product is exactly equal to his/her reservation price

A.2 General Personal Information

1. How hungry are you right now?
   (Not hungry at all) 0, 1 . . . , 10 (Very hungry)

2. When did you have your last meal?

   (a) Within the last 30 mins

   (b) 30 mins - 2 hours ago

   (c) 2 hours - 6 hours ago

   (d) 6 hours - 12 hours ago

   (e) 12 hours - 24 hours ago

   (f) More than 24 hours ago

3. When did you have your last snack?

(a) Within the last 30 mins
(b) 30 mins - 2 hours ago
(c) 2 hours - 6 hours ago
(d) 6 hours - 12 hours ago
(e) 12 hours - 24 hours ago
(f) More than 24 hours ago

4. Are you on diet right now?
   (a) Yes
   (b) No
5. How motivated are you with your diet goal?
   (Not motivated at all) 0, 1 . . . , 10 (Extremely motivated)
6. How far are you from your diet goal?
   (Just started) 0, 1 . . . , 10 (Almost done)

A.3 Chocolate-Specific Questions

We ask this set of questions for each of the four kinds of chocolates respectively.

1. How familiar are you with this brand?
   (Not at all) 0, 1 . . . , 10 (Very familiar)
2. How much do you like this brand?
   (Not at all) 0, 1 . . . , 10 (Very much)
3. How prestigious is this brand, relative to all the chocolate brands you know?
   (Not prestigious at all) 0, 1 . . . , 10 (Very prestigious)
4. How often do you purchase product from this brand?
   (a) Never
   (b) Less than Once a Month
   (c) Once a Month
   (d) 2-3 Times a Month
   (e) Once a Week
   (f) 2-3 Times a Week
   (g) Daily
5. How much do you like this flavour?
   (Not at all) 0, 1 . . . , 10 (Very much)
6. How much do you like the wrapping package of the chocolate?
   (Not at all) 0, 1 . . . , 10 (Very much)
7. How much do you like this chocolate overall?
   (Not at all) 0, 1 . . . , 10 (Very much)
8. What is the likelihood for you to buy it in the next week, if it is available in a nearby store?
   (Not likely at all) 0, 1 . . . , 10 (Extremely likely)
9. How likely will you recommend this chocolate to your friends?
   (Not likely at all) 0, 1 . . . , 10 (Extremely likely)
10. How tasty do you believe the chocolate is?
    (Not at all) 0, 1 . . . , 10 (Very much)
11. How much do you want to eat this chocolate right now?
    (Not at all) 0, 1 . . . , 10 (Very much)
12. What is the brand name of the chocolate you just saw?
    (Attention check question)
13. What is your reservation price for this chocolate right now?
    (Definition of RP shows up here again)
14. For each price listed below, please indicate if you would like to buy the chocolate at that price or not. For each price, you must choose either of the two options.

At price	will NOT buy the chocolate	will buy the chocolate
\$13.00	☐	☐
\$12.50	☐	☐
. . .	. . .	. . .
\$1.00	☐	☐
\$0.50	☐	☐

A.4 General Questions About Chocolate

1. How often do you buy chocolate?
   (a) Never
   (b) Less than Once a Month
   (c) Once a Month
   (d) 2-3 Times a Month
   (e) Once a Week
   (f) 2-3 Times a Week
   (g) Daily
2. How much do you like eating chocolate?
   (Not at all) 0, 1 . . . , 10 (Very much)
3. What kind of features of chocolate do you like?
   (a) high percentage of cocoa [0-100]
   (b) white chocolate [0-100]
   (c) with milk [0-100]
   (d) with nuts [0-100]
   (e) fruit flavor [0-100]
   (f) exotic flavor (e.g., spicy) [0-100]
4. On average, how much do you pay on chocolate in each grocery shopping?
5. For all the possible chocolates available (weight: 100g), what is the highest price you are willing to pay?
6. When making chocolate purchase decisions, how important is the [] of chocolate on affecting your decision?
   (a) price [0-100]
   (b) size/weight [0-100]
   (c) cocoa percentage [0-100]
   (d) origin [0-100]
   (e) shape&looking [0-100]
   (f) fair trade [0-100]
7. How much concerns do you have with your body-image?
   (Not at all) 0, 1 . . . , 10 (Very much)
8. How often do you calculate food calories?
   (Never) 0, 1 . . . , 10 (Often)
9. How much control do you have on your daily sugar-ingestion?
   (No control at all) 0, 1 . . . , 10 (rigorous control)
10. How often do you consume snacks?
    (a) Never
    (b) Less than Once a Month
    (c) Once a Month
    (d) 2-3 Times a Month
    (e) Once a Week
    (f) 2-3 Times a Week
    (g) Daily

A.5 Demographics Questions

1. What is your age?
2. In which country do you reside?
3. What is your gender?
4. What is your employment status?
5. What is your monthly household income?

A.6 Final Attention Check Question

1. Which of the following brands appeared in this survey? (You can check more than one option)
   (a) Lindt douban
   (b) M&M
   (c) Godiva
   (d) Leonidas

B Detailed Empirical Results

Table 6. Each cell gives the MAE "mean (std dev)" over the ten-times ten-fold cross validation, of running a particular learner on the dataset, broken out by the brand of chocolate. **Bold** values are the best performance across the five models for that dataset. Table 7 uses the same format.

Brand	Godiva	Lindt	Valrhona	Hersheys	**Overall**
Baseline	2.14 (0.26)	1.40 (0.14)	1.55 (0.18)	0.72 (0.09)	1.45 (0.09)
KM	2.13 (0.26)	1.39 (0.14)	1.53 (0.18)	0.73 (0.09)	1.44 (0.09)
AFT	1.81 (0.21)	1.30 (0.15)	**1.22** (0.15)	**0.62** (0.07)	1.24 (0.08)
Cox	**1.77** (0.21)	1.29 (0.15)	1.23 (0.15)	0.63 (0.08)	**1.23** (0.08)
MTLR	1.80 (0.22)	**1.24** (0.14)	**1.22** (0.14)	0.68 (0.09)	**1.23** (0.08)

Table 7. Classification accuracy (ten-times ten-fold cross validation).

Brand	Godiva	Lindt	Valrhona	Hersheys	**Overall**
Baseline	0.53 (0.02)	0.54 (0.03)	0.54 (0.02)	0.55 (0.04)	0.54 (0.01)
KM	0.74 (0.04)	0.77 (0.06)	0.76 (0.06)	0.75 (0.03)	0.76 (0.02)
AFT	**0.80** (0.03)	**0.80** (0.03)	0.81 (0.04)	**0.81** (0.05)	**0.80** (0.02)
Cox	0.79 (0.04)	**0.80** (0.02)	**0.82** (0.04)	0.80 (0.04)	**0.80** (0.01)
MTLR	**0.80** (0.04)	**0.80** (0.05)	**0.82** (0.05)	0.79 (0.04)	**0.80** (0.02)

Table 8. Profit values for each cost of production – corresponding to Figure 11.

Cost	Retail	LR	LDA	NB	KM	AFT	Cox	MTLR
0.10 s_ω	1.44	1.41	1.31	1.03	1.43	1.44	1.51	**1.52**
0.15 s_ω	1.36	1.31	1.22	0.97	1.34	1.35	1.42	**1.43**
0.20 s_ω	1.28	1.22	1.13	0.91	1.25	1.25	**1.32**	**1.32**
0.25 s_ω	1.20	1.13	1.06	0.85	1.17	1.15	**1.23**	**1.23**
0.30 s_ω	1.12	1.05	0.97	0.79	1.07	1.06	**1.14**	**1.14**
0.35 s_ω	1.04	0.97	0.90	0.72	0.97	0.98	1.04	**1.05**
0.40 s_ω	**0.96**	0.89	0.83	0.67	0.86	0.90	**0.96**	**0.96**
0.45 s_ω	**0.88**	0.82	0.76	0.62	0.77	0.82	0.87	**0.88**
0.50 s_ω	0.80	0.75	0.69	0.58	0.69	0.74	0.79	**0.81**
0.55 s_ω	0.72	0.68	0.63	0.53	0.63	0.68	0.72	**0.73**
0.60 s_ω	0.64	0.62	0.57	0.49	0.57	0.61	0.65	**0.66**
0.65 s_ω	0.56	0.56	0.52	0.45	0.51	0.55	0.59	**0.60**
0.70 s_ω	0.48	0.50	0.47	0.41	0.46	0.49	0.53	**0.54**
0.75 s_ω	0.40	0.46	0.41	0.37	0.41	0.43	**0.48**	**0.48**
0.80 s_ω	0.32	0.41	0.37	0.34	0.37	0.38	**0.43**	**0.43**
0.85 s_ω	0.24	0.36	0.33	0.31	0.33	0.34	**0.39**	0.38
0.90 s_ω	0.16	0.32	0.29	0.28	0.30	0.31	**0.34**	**0.34**

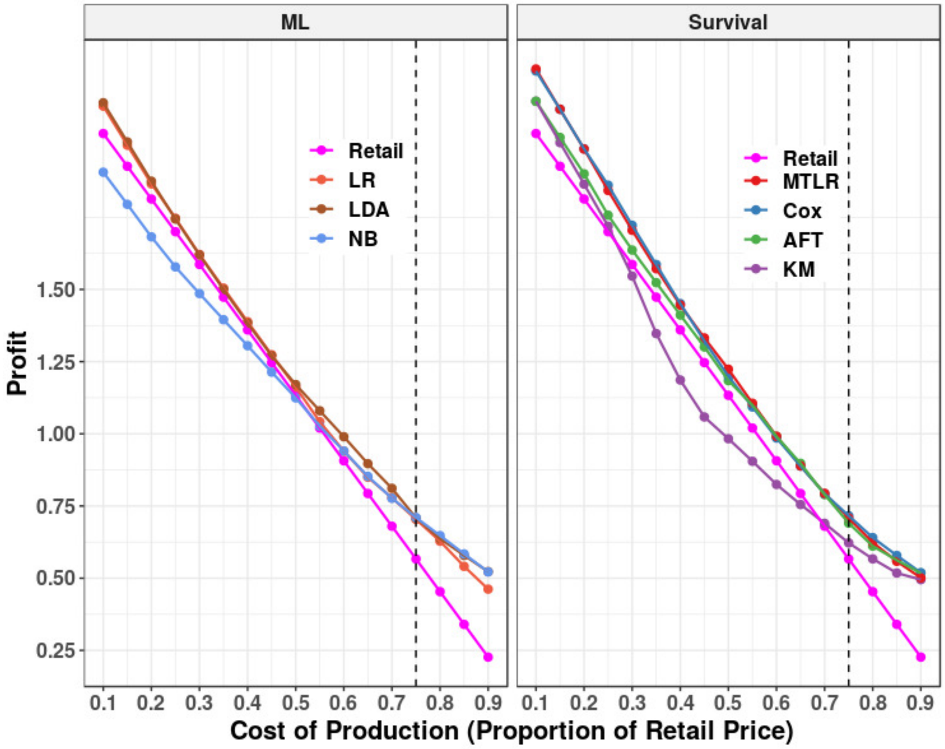

Fig 12. Average profit generated from Godiva chocolate: 10-times 10-fold cross validation results.

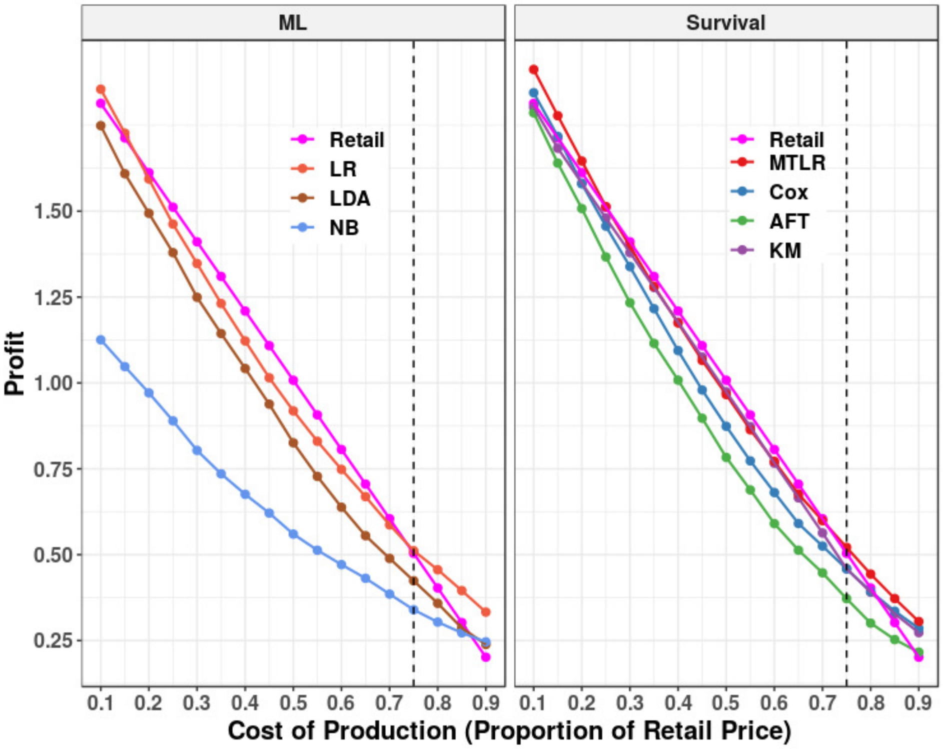

Fig 13. Average profit generated from Lindt chocolate: 10-times 10-fold cross validation results.

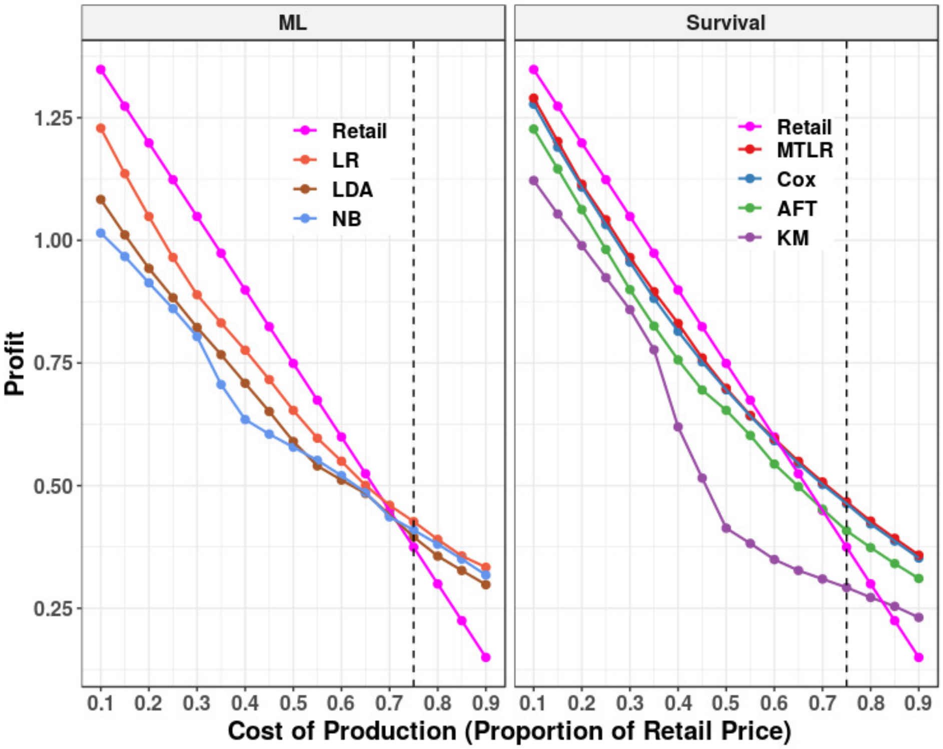

Fig 14. Average profit generated from Valrhona chocolate: 10-times 10-fold cross validation results.

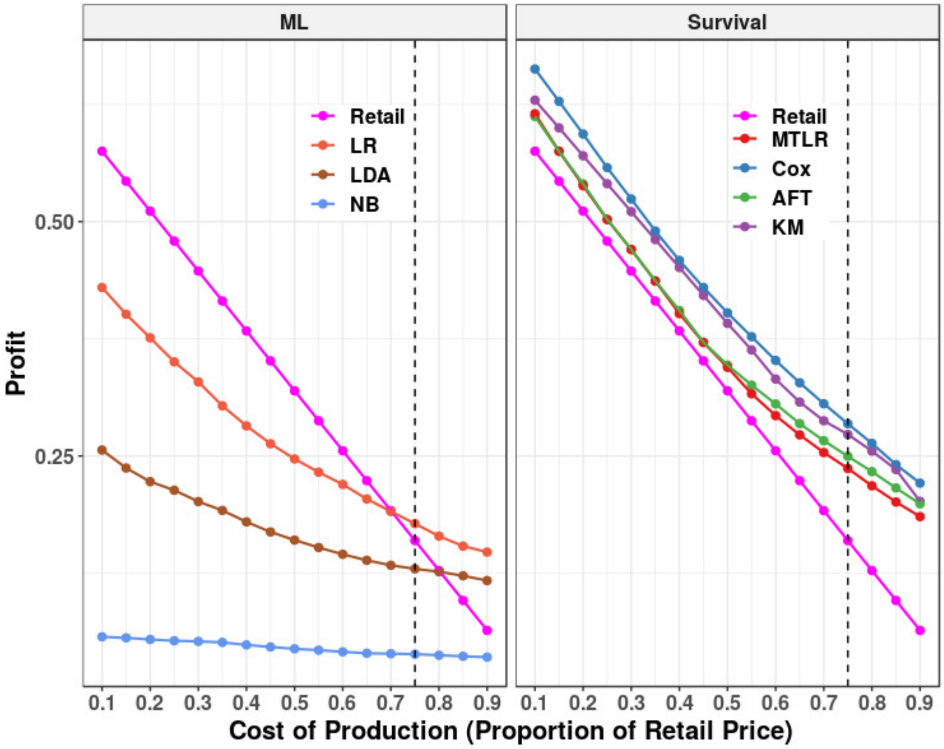

Fig 15. Average profit generated from Hershey's chocolate: 10-times 10-fold cross validation results.
